# Dynamic Dependence on ATR and ATM for Double-Strand Break Repair in Human Embryonic Stem Cells and Neural Descendants

**DOI:** 10.1371/journal.pone.0010001

**Published:** 2010-04-02

**Authors:** Bret R. Adams, Sarah E. Golding, Raj R. Rao, Kristoffer Valerie

**Affiliations:** 1 Department of Radiation Oncology, Virginia Commonwealth University, Richmond, Virginia, United States of America; 2 Department of Biochemistry and Molecular Biology, Virginia Commonwealth University, Richmond, Virginia, United States of America; 3 Department of Chemical and Life Sciences Engineering, Virginia Commonwealth University, Richmond, Virginia, United States of America; 4 The Massey Cancer Center, Virginia Commonwealth University, Richmond, Virginia, United States of America; University of Minnesota, United States of America

## Abstract

The DNA double-strand break (DSB) is the most toxic form of DNA damage. Studies aimed at characterizing DNA repair during development suggest that homologous recombination repair (HRR) is more critical in pluripotent cells compared to differentiated somatic cells in which nonhomologous end joining (NHEJ) is dominant. We have characterized the DNA damage response (DDR) and quality of DNA double-strand break (DSB) repair in human embryonic stem cells (hESCs), and *in vitro*-derived neural cells. Resolution of ionizing radiation-induced foci (IRIF) was used as a surrogate for DSB repair. The resolution of γ-H2AX foci occurred at a slower rate in hESCs compared to neural progenitors (NPs) and astrocytes perhaps reflective of more complex DSB repair in hESCs. In addition, the resolution of RAD51 foci, indicative of active homologous recombination repair (HRR), showed that hESCs as well as NPs have high capacity for HRR, whereas astrocytes do not. Importantly, the ATM kinase was shown to be critical for foci formation in astrocytes, but not in hESCs, suggesting that the DDR is different in these cells. Blocking the ATM kinase in astrocytes not only prevented the formation but also completely disassembled preformed repair foci. The ability of hESCs to form IRIF was abrogated with caffeine and siRNAs targeted against ATR, implicating that hESCs rely on ATR, rather than ATM for regulating DSB repair. This relationship dynamically changed as cells differentiated. Interestingly, while the inhibition of the DNA-PKcs kinase (and presumably non-homologous endjoining [NHEJ]) in astrocytes slowed IRIF resolution it did not in hESCs, suggesting that repair in hESCs does not utilize DNA-PKcs. Altogether, our results show that hESCs have efficient DSB repair that is largely ATR-dependent HRR, whereas astrocytes critically depend on ATM for NHEJ, which, in part, is DNA-PKcs-independent.

## Introduction

Human embryonic stem cells (hESCs) possess the unique characteristic of indefinite self-renewal while remaining in an undifferentiated state. Possible reasons for this include the ability to maintain telomere length, avoid senescence through a non-functional p53 pathway, and preserve genomic and epigenetic integrity to a higher degree than somatic cells [1], [2]. Maintaining genomic integrity is vital for the stem cell because mutations will severely compromise all derived cell lineages and their progenies. A stable genome requires the coordination of multiple processes referred to as the DNA damage response (DDR), including cell cycle checkpoints, DNA repair, and apoptosis [3]. Several studies support the hypothesis that as mouse embryonic stem cells differentiate DNA repair is down-regulated and the genomic integrity of surviving ESCs is preserved by hypersensitivity to DNA damaging agents and high levels of cell death [1], [4]. On the other hand, other studies suggest there is increased repair in ESCs with homologous recombination repair (HRR) favored relative to non-homologous end joining (NHEJ) early in mouse development [5], [6]. Little is known about these processes in hESCs and in vitro-derived neural cells and it is almost certain that differences between the human and mouse systems exists [7], [8].

The most detrimental form of DNA damage is the double-strand break (DSB), which is repaired primarily by NHEJ, and to a lesser extent by HRR in human cells. NHEJ is a potentially error-prone form of repair that is initiated with the binding of the KU70/KU80 heterodimer to DNA ends which then recruits the catalytic subunit of DNA-dependent protein kinase (DNA-PKcs). The ends are then resected by the Artemis and/or MRE11/RAD50/NBS1 (MRN) nucleases, followed by XRCC4, XLF, and DNA ligase IV recruitment necessary for resealing [3]. HRR uses a DNA template, either in the form of a sister chromatid, homologous chromosome, or repeated sequence, to ensure high-fidelity repair. This process involves BRCA2 and BRCA1, RAD51 and a group of RAD51-related proteins, and the MRE11/RAD50/NBS1 complex [9]. NHEJ occurs throughout the cell cycle but predominates in G1, whereas HRR primarily occurs during late S and G2 phases when sister chromatids are available [10].

The major DNA damage sensors are ataxia telangiectasia mutated (ATM) and ataxia telangiectasia and RAD3-related (ATR) that coordinate the DDR and help ensure the preservation of the genome [3], [11]. Mutation or loss of ATM or ATR lead to cell senescence, accelerated aging, and premature death associated with increased genomic instability [12], [13]. One of the first events marking the DSB is the phosphorylation of the histone H2A isoform H2AX at S139, also referred to as γ-H2AX, which is mediated by either ATM, ATR, or DNA-PKcs [14], [15], [16]. The formation and resolution of γ-H2AX is linked to the presence of DSBs and can act as a surrogate for DNA damage and DSB repair [14], [17]. Following the phosphorylation of H2AX, repair proteins such as the MRN complex, ATM, and 53BP1 coordinate cell cycle arrest, DNA repair and apoptosis [3]. Whereas the resolution of γ-H2AX and 53BP1 ionizing radiation-induced foci (IRIF) are not specific for either HRR or NHEJ, RAD51 is uniquely associated with HRR [9].

Herein, we show that by directing hESCs down a specific neural cell lineage *in vitro* to a differentiated, mitotically inactive state, dynamic changes in DSB repair are revealed. ATM has traditionally been associated with the DDR and repair of DSBs induced by radiation. Here we show that in hESCs ATR is taking ATM's role as the primary PIKK. We also show that ATM is critical for DSB repair in astrocytes, and have begun defining the role of ATR in DSB repair in hESCs.

## Results

### DSB repair in hESCs, neural progenitors and astrocytes

**Figure 1:**
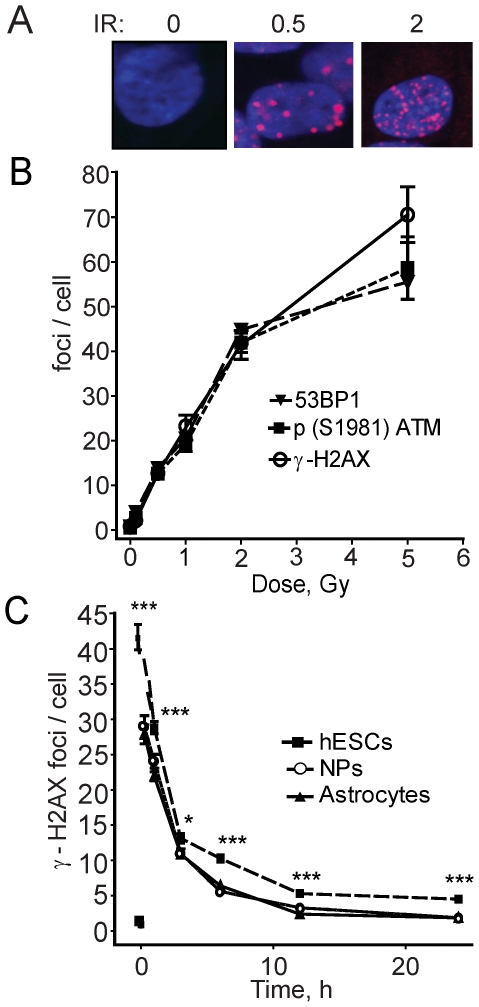
Characterization of repair foci formation and resolution in hESCs, neural progenitors and astrocytes. **A**. Depicted is immunostaining of hESCs after treatment with 0, 0.5, and 2 Gy and exposed to p-(S1981) ATM antibody 15 min after radiation. **B**. Immunostaining of 53BP1, p-(S1981) ATM, and γ-H2AX foci 15 min after irradiation in hESCs after doses from 0.1 to 5 Gy. n = 30 cells per time point. **C**. Graphical depiction of the number of γ-H2AX foci in hESCs, NPs and astrocytes exposed to 2 Gy over 24 h. Data are expressed as mean IRIF per cell. n = 100 cells per time point. There was <1 focus on average in the untreated hESC, NP and astrocyte populations (shown as symbols at time 0). *Error bars* indicate the standard error of the mean (SEM). Asterisks indicate statistical significance between the number of foci observed in hESCs, NPs, and astrocytes at the same time point.

Our previous work established optimal conditions for the growth and differentiation of hESCs into neural progenitors (NPs) and astrocytes (Figure S1) [18]. Importantly, since neural descendants are all derived from the same parental embryonic cells any alteration in phenotype must be due to changes in the epigenetic control of the cells. Importantly, we grow the hESCs on an extra-cellular substrate without a MEF feeder layer to avoid contamination of the hESCs with mouse cells [19]. Immunoflourescence and IRIF assays were used to monitor surrogate markers of DSBs in the different cell populations. An example of hESCs exposed to radiation at low doses and subsequent foci formation and resolution is shown (Figure 1). The number of p-ATM, γ-H2AX and 53BP1 foci increased in a dose-dependent manner from a dose as low as 0.1 Gy, and this response was linear between 0.1 and 2 Gy (Figure 1A and B).

Then, hESCs, NPs, and astrocytes were examined for their capacity to repair DSBs by following the resolution of γ-H2AX foci over time (Figure 1C). hESCs showed a significantly greater number of γ-H2AX foci 15 min after irradiation compared to NPs and astrocytes. The mean focus size was also smaller in the hESCs than in the NPs or astrocytes. Furthermore, hESCs had significantly greater numbers of IRIF (30–45%) remaining at later time points compared to NPs and astrocytes (Figure 1C). Thus, this difference in the rate of resolution may reflect a change in the quality of DSB repair with more complex and slower repair in hESCs than in NPs and astrocytes.

### hESCs predominantly use HRR whereas astrocytes lack HRR

**Figure 2:**
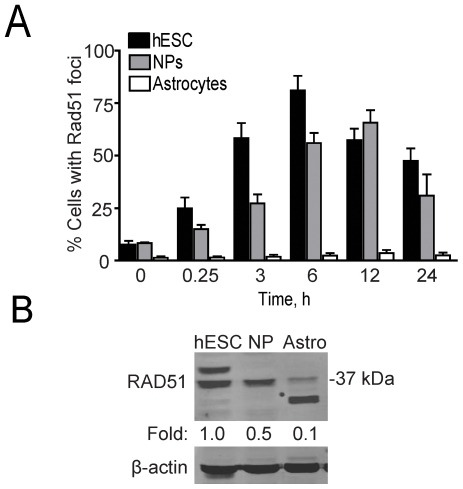
Differentiation affects RAD51 foci formation and expression. **A**. Graphical depiction of the percentage of RAD51 foci in hESCs, NPs, and astrocytes after 2 Gy. Cells were scored either as positive (>1 focus) or negative (≤1 foci) for RAD51. *Errors bars*, SEM for three independent data sets with n>200. There were statistically significant (p<0.05) increases in the number of cells with RAD51 foci after radiation compared to unirradiated cells at all time points for hESCs and NPs whereas there was no statistical change in the astrocytes. **B**. Western blot showing expression of RAD51 in hESC, NPs and astrocytes. Fold depicts relative differences in RAD51 levels of the 37-kDa form after normalization to β-actin which served as a loading control.

HRR represents high-fidelity DSB repair occurring mainly in late S and G2 of the cell cycle [20]. The ability of cells to undergo HRR is uniquely dependent on RAD51, which is involved in the search for DNA homology and in the strand-pairing stages [3]. Thus, as a surrogate marker for HRR, we examined the formation and resolution of RAD51 foci after radiation. hESCs and NPs showed significant increases in the number of cells with RAD51 foci as early as 15 min after radiation (Figure 2). Peak levels (>75%) occurred after 6 h in hESCs whereas in NPs levels peaked at 12 h with ∼65% of the cells having RAD51 foci (Figure 2A). Conversely, astrocytes showed little to no cells with RAD51 foci (<3%) after radiation in the same time window suggesting that these cells completely lack HRR as expected.

A direct correlation between the percentage of cells having RAD51 foci with RAD51 protein levels was noted (Figure 2B). The reported molecular mass of human RAD51 is ∼37-kDa, which diminished as the hESCs differentiated into NPs and astrocytes (1, 0.5, and 0.1-fold, respectively) correlating well with the RAD51 foci result (see Figure 2A). In addition, RAD51 might exist in different forms in the three cell populations. A ∼41-kDa protein species in addition to the 37-kDa form appeared in the extract from hESCs, and a band at ∼31-kDa was evident in the extract from the astrocytes (Figure 2B). These data indicate that hESCs and NPs rely on HRR while differentiated astrocytes have much reduced RAD51 levels (37-kDa form), show no RAD51 foci, and, thus, lack HRR.

### ATM is dispensable for the formation and resolution of DSBs in hESCs but is indispensible in astrocytes

**Figure 3:**
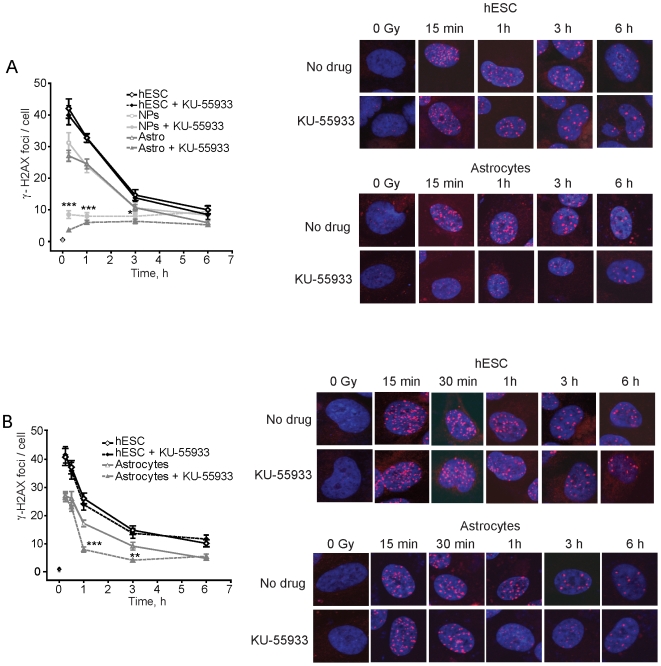
ATM kinase is important for γ-H2AX foci resolution in astrocytes but not in hESCs. Graphical depiction (left panel) and images (right panel) of γ-H2AX foci in hESCs, NPs, and astrocytes exposed to 10 µM KU-55933 **A**. 1 h prior to or **B**. 15 min after radiation with 2 Gy. There was <1 focus on average in the untreated hESC, NP and astrocyte populations (shown as symbols at time 0). Asterisks indicate statistical significance between the data sets generated with NPs and astrocytes, respectively, with KU-55933 present or not at the same time point. *Data points*, foci per nucleus; *Error bars*, SEM for data sets n = 50.

ATM regulates the DDR after radiation and was shown to be critical for both HRR and NHEJ in human cells [21], [22]. To determine whether ATM plays a role in the resolution of IRIF in hESCs, NPs, and astrocytes, we used a highly specific ATM kinase inhibitor, KU-55933 [21], [23], [24]. There was no difference in the resolution of γ-H2AX IRIF between untreated and KU-55933-treated hESCs indicating that ATM is dispensable for DSB repair in these cells (Figure 3A). On the other hand, both NPs and astrocytes responded as expected based on our previous results with human glioma cells [24]. γ-H2AX foci formation after irradiation was reduced 70–85% in the KU-55933-treated cells compared to the untreated controls, in sharp contrast to the result with the hESCs (Figure 3A). Similar results were obtained when p(S1981)-ATM foci were examined (data not shown). The finding that there was no effect of KU-55933 on DSB repair in BG01V cells was confirmed with the H9 hESCs suggesting that this result was not an anomaly associated with the BG01V cells (Figure S2).

Not only did IRIF formation not occur in astrocytes treated with KU-55933 prior to radiation, but the drug also promoted the disassembly of already formed foci (Figure 3B). The sharp increase in IRIF formation observed at early times (<3 h) was not seen and foci in irradiated cells treated with KU-55933 became more diffuse and completely dissolved over time. Thus, the apparent disappearance of γ-H2AX foci in the presence of KU-55933 is likely due to the disassembly of repair centers and not more proficient repair, as suggested by the steeper slope of the curve for KU-55933-treated astrocytes (Figure 3B).

**Figure 4:**
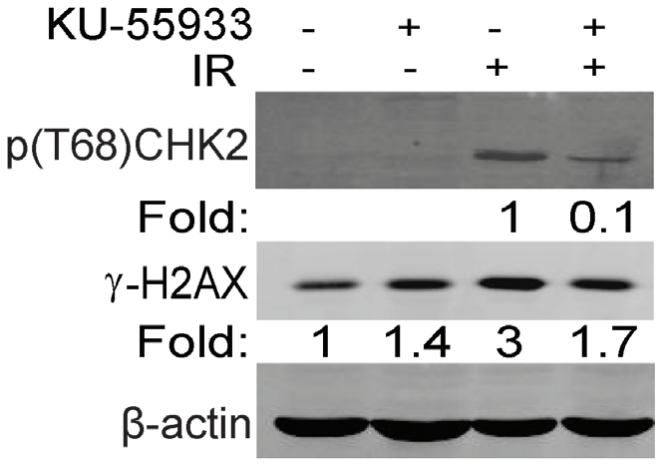
ATM kinase is functioning in hESCs. Western blot of extracts isolated from hESCs 15 min after exposure to 5 Gy with or without KU-55933 (10 µM) added 1 h prior to radiation. Fold change depicts phosphorylation of CHK2 (T68) and H2AX (S139) after normalization to β-actin which served as a loading control.

A possible explanation for the lack of an effect of KU-55933 on the resolution of radiation-induced γ-H2AX foci in hESCs could be that these cells were refractory to the drug (poor uptake, more effective drug break-down, etc) or, alternatively, they do not depend on ATM for DSB repair. To distinguish between these two possibilities, we examined the effect of KU-55933 on preventing the phosphorylation of CHK2 at T68 in irradiated hESCs. CHK2 phosphorylation at this position is believed to be largely ATM-dependent [23], [25]. Surprisingly, we found that when KU-55933 was used at a dose that blocks radiation-induced phosphorylation of p53 (S15), H2AX (S139), and CHK2 (T68) in an ATM-dependent manner in human tumor cell lines [24], it did in fact inhibit CHK2 phosphorylation >85% in irradiated hESCs (Figure 4). Similarly, γ-H2AX levels were reduced ∼50% when cells were exposed to KU-55933. Therefore, the lack of an effect of KU-55933 in hESCs by IRIF assay is not because the drug does not work or that the ATM kinase is not available or active in these cells, but instead because ATM is not critical for the formation and resolution of repair foci in hESCs.

### Blocking ATR expression abrogates DSB repair in hESCs

**Figure 5:**
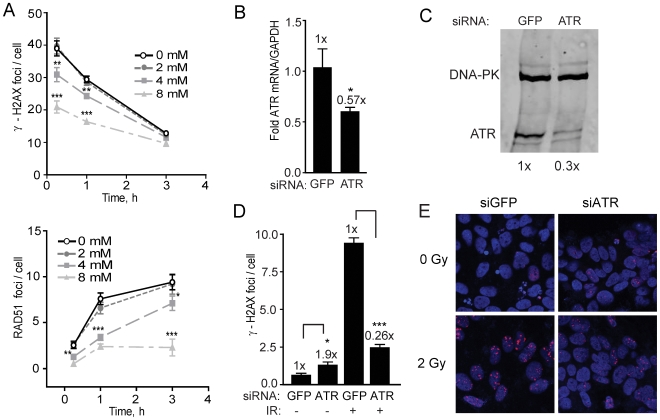
ATR is required for DSB repair in hESCs. **A**. Graphical depiction of γ-H2AX (top panel), and RAD51 (bottom panel) foci determined in the presence of 0, 2, 4, and 8 mM caffeine administered 1 h prior to radiation (2 Gy). Asterisks designate statistical significance between the two data sets adjacent to the asterisk at the same time point. *Data points*, foci per nucleus; *Error bars*, SEM for data sets n = 50. **B**. Graphical depiction of ATR mRNA levels normalized to GAPDH mRNA after transfection with GFP control siRNAs and siRNAs targeting ATR. Experiments were performed in triplicate, and the SEM indicated by error bars. **C**. Western blot showing ATR expression 48 h after transfection of BGO1V cells with GFP control siRNAs or siRNAs targeting ATR. The fold change in ATR was calculated after normalization to DNA-PKcs which served as a loading control. **D**. Graphical depiction of the effects of ATR knockdown on DSB repair determined by γ-H2AX foci. Cells were transfected with GFP control siRNAs or siRNAs targeting ATR, irradiated with 2 Gy 48 h after transfection, and fixed 6 h later. *Data points*, γ-H2AX foci per nucleus; *Error bars*, SEM for data sets n = 100. **E**. Representative images of cells with γ-H2AX foci (red) with DAPI staining showing nuclei (blue).

ATR is known to serve as backup for ATM, is critical for regulating the DDR during replication, and is important for DSB repair [26], [27]. Caffeine inhibits both ATM and ATR, with a greater potency against ATM than ATR [28]. Thus, caffeine was first used to examine the effects on γ-H2AX foci formation after irradiation. At 2 mM caffeine is expected to inhibit ATM but not ATR [29]. Indeed, at this concentration there was no effect on γ-H2AX foci formation (Figure 5A), a result in agreement with the finding that KU-55933 does not affect foci formation in these cells (see Figure 3). However, after treatment with 4 and 8 mM, 21% and 47% reduction in γ-H2AX foci, respectively, was observed at early times after irradiation (Figure 5A, top panel). Furthermore, RAD51 foci were almost completely absent in cells treated with 8 mM caffeine (≤3 h) and significantly reduced in cells treated with 4 mM (Figure 5A, bottom panel). Later time points (>3 h) showed significant cell death with both concentrations of caffeine, suggesting that inhibition of ATM and ATR is very toxic to hESCs.

To confirm the result with caffeine and more clearly establish a role for ATR in DSB repair in hESCs, we transfected the cells with siRNA targeting ATR and examined the impact on DSB repair. hESCs transfected with ATR siRNAs showed significant (45–70%) knockdown of ATR expression, but no effect on DNA-PKcs levels (Figure 5B and C). This knockdown led to a reduction of γ-H2AX foci by >70% in cells treated with radiation (Figure 5D and E). Interestingly, unirradiated cells showed low levels of γ-H2AX foci, which increased almost 2-fold when ATR was knocked down. These results suggest that ATR is critical for HRR in hESCs.

### Astrocytes require ATM, and in part DNA-PKcs, for DSB repair

**Figure 6:**
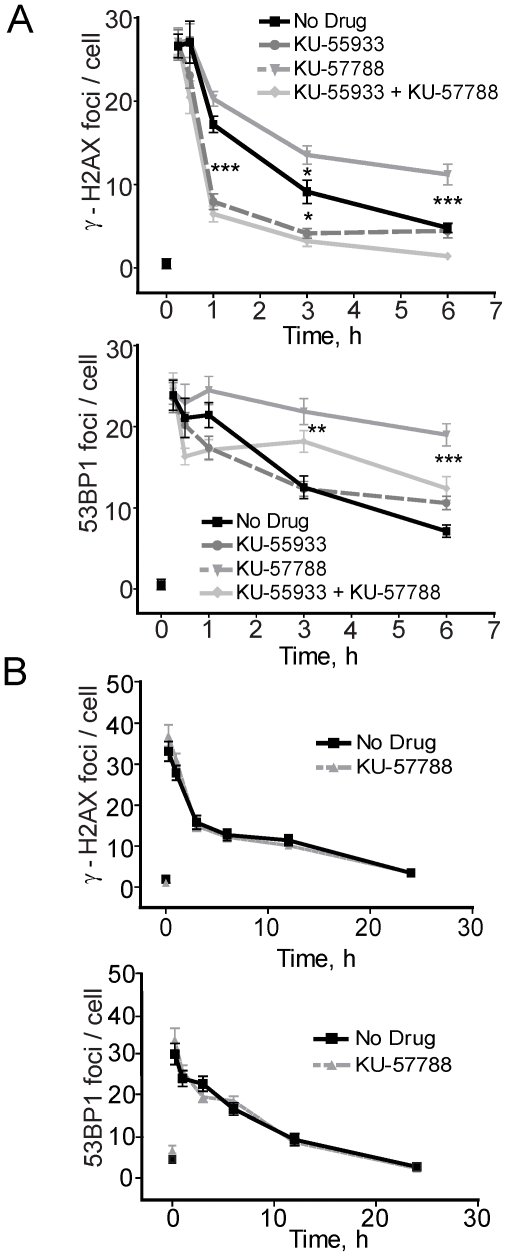
Astrocytes rely completely on NHEJ. **A**. Graphical depiction of γ-H2AX (top panel) and 53BP1 (bottom panel) foci in astrocytes after radiation. Cells were treated or not with KU-55933 (10 µM), KU-57788 (2.5 µM) or both for 1 h followed by irradiation with 2 Gy. **B**. Graphical depiction of γ-H2AX (top panel) and 53BP1 (bottom panel) foci in BGO1V cells treated or not with KU-57788 (2.5 µM) followed by radiation with 2 Gy. Asterisks designate statistical significance between data sets of untreated and drug-treated cells at the same time point. There was <1 focus on average in the untreated hESC, NP and astrocyte populations. *Data points*, foci per nucleus; *Error bars*, SEM for data sets n = 50.

To further characterize DSB repair in the astrocytes, we examined a possible dependence on DNA-PKcs in the repair of DSBs. Taking advantage of the highly specific DNA-PKcs kinase inhibitor, KU-57788 (NU7441) [24], [30], [31], we show that DNA-PKcs, in part, is important for the resolution of both γ-H2AX and 53BP1 foci in astrocytes (Figure 6A). At 6 h after irradiation, >40% of γ-H2AX foci remained in the KU-57788-treated astrocytes compared to only 21% in the untreated. Importantly, the KU-57788-treated cells had more repair foci remaining throughout the entire time course, suggesting that DNA-PKcs is important for repair but is not fully dependent on DNA-PKcs. This response is in sharp contrast to that seen when cells were treated with KU-55933 where the slope was steeper, most likely reflecting the disassembly of repair complexes (see Figure 3). Similarly, ∼80% of 53BP1 foci remained in the KU-57788-treated cells compared to only ∼30% in the untreated cells when the drug was added after irradiation (Figure 6A, bottom panel). These results further suggests that repair complexes remain at the DSB but repair proceeds slower, reflecting a dependence (50–65%) on DNA-PKcs, presumably as a critical partaker in NHEJ [3]. Importantly, when both KU-57788 and KU-55933 were combined, there was a complete lack of γ-H2AX foci formation after irradiation, demonstrating an effect similar to KU-55933 alone (Figure 6A).

Whereas hESCs depend predominantly on HRR, as we have shown here, there was no effect of KU-57788 on the resolution of either γ-H2AX or 53BP1 IRIF in hESCs (Figure 6B), a result which is in sharp contrast to that seen with the astrocytes. This result was also recapitulated with the H9 hESC line (Figure S2). This result suggests that DNA-PKcs is not important for DSB repair in hESCs.

## Discussion

It is important to understand the cellular responses occurring in normal brain during chemo- and radiotherapy, to counter possible adverse effects, and increase the therapeutic ratio to improve the treatment of brain cancer. Neural cells derived from hESCs provide an excellent in vitro experimental system for studying the DDR in normal human brain. It is equally important to fully comprehend how genomic stability can be preserved in hESCs during in vitro propagation for future, safe applications in regenerative medicine. Herein, we show that the cellular response to DSBs changes as hESCs differentiate into non-cycling astrocytes, and have begun to characterize this dynamic relationship as it relates to the dependence of DNA damage sensors and effects on DSB repair.

Current understanding of the DDR in hESCs is that these cells have a reduced G1 phase and may lack the G1/S checkpoint altogether [4], [32]. Glioma cells show delayed resolution of γ-H2AX foci at later times after irradiation if they lack p53 and, hence, the G1/S checkpoint [33]. Thus, one explanation for the slow resolution of foci in hESCs may be more robust cell cycle checkpoints (other than G1/S) and slower DDR recovery [34]. Our results are consistent with previous reports suggesting that hESCs have a robust and active G2/M checkpoint and an absent G1/S checkpoint, which first appears in NPs after differentiation of hESCs [4], [32], [35]. A recent report showed that KU-55933 was ineffective at inhibiting the G2 checkpoint after irradiation in hESCs unless extremely high concentrations were used despite the fact that phosphorylation of T68-CHK2, S15-p53 were significantly reduced with 10 µM [36]. We show that KU-55933 is effective in hESCs at lower concentrations typically used to block the DDR in tumor cell lines since radiation-induced CHK2 and H2AX phosphorylation was inhibited [24]. This consistent effect of KU-55933 on hESC would thwart the notion of any significant effect of a drug pump [36]. The reason why we do not see any effect of KU-55933 on the formation of p-(S1981)-ATM foci after radiation of hESCs is most likely because this antibody recognizes a number of other DDR proteins phosphorylated by ATM and ATR, such as SMC1, in addition to auto-phosphorylated ATM [37]. If ATR takes ATM's place SMC1 and other targets would still be phosphorylated. Thus, ATM seems to play a role in hESCs but does not affect DSB repair, suggesting that in these rapidly proliferating cells another PIKK besides ATM is responsible for regulating DSB repair. ATR is a likely candidate as we have shown herein. Recently, through genetic manipulation by targeted allele disruption an ATM knockout hESC line was established [38]. These cells demonstrated a reduction of ATM downstream signaling after radiation including reduced phosphorylation of H2AX (S139), and CHK2 (T68), but no genetic instability by CGH analysis. Thus, these results are in line with our finding that ATM does not play a critical role in DSB repair in hESCs.

The initial number of γ-H2AX foci after irradiation was greater in hESCs compared to those in either NPs or astrocytes. Previously, when the cell cycle was correlated with the size of repair foci, it was found that irradiation of S-phase cells generated a greater number of smaller IRIF believed to represent a collapse of the replication machinery at sites of single-strand breaks [39]. Thus, because a large fraction of hESCs is in S-phase at a given time, it is possible that a larger number of foci are seen in these cells compared to NPs and astrocytes. Furthermore, later times after irradiation showed more residual foci in hESCs in our study. The most reasonable explanation for this finding is that these cells depend largely on slower, more complex, DSB repair than either NPs or astrocytes.

Our results show that HRR is highly active in hESCs and declines as cells differentiate into NPs, and disappear entirely after differentiation into astrocytes. hESCs display a higher percentage of cells with RAD51 foci at early times after irradiation when compared to NPs, indicating more robust HRR, whereas in astrocytes, no RAD51 foci were seen. In support of this finding, we found that the expression of the 37-kDa form of RAD51 gradually decreased along the hESC-NP-astrocyte axis. Furthermore, a larger form of RAD51 was found in hESCs, which could be the result of alternative splicing, and RAD51 in astrocytes seems to undergo post-translational processing, both of which could affect HRR. A previous report showed that cleavage of RAD51 to a 31-kDa protein occurs in cells undergoing apoptosis [40], [41]. Thus, the smaller RAD51 species seen in the extract from astrocytes could perhaps be an inactive form of RAD51. Altogether, the level of full-length RAD51, and possible RAD51 processing could perhaps represent different layers of HRR control in these cell populations.

Previous work established that ATM is inhibited by caffeine more readily than ATR [28], [29]. We show that caffeine at doses ≥4 mM inhibit IRIF formation in hESCs. In line with this finding, knockdown of ATR reduced γ-H2AX and RAD51 IRIF supporting the idea that ATR is critical for HRR in hESCs. Furthermore, we noticed a significant increase in the level of γ-H2AX foci in unirradiated cells after ATR knockdown which seems to also occur in ATR-deficient mouse cells due to increased replicative stress induction of DSBs [13]. If hESCs only require ATR but not ATM for DSB repair what could be the role of ATM in these cells? It is possible that ATR-dependent HRR is so dominant in hESCs compared to ATM-dependent repair and thus cannot be detected. It is also possible that ATM kinase targets, such as CHK2 and p53, are either sequestered or not fully functioning in hESCs [2]. ATM could also be critical for apoptosis and for maintaining the progenitor cell phenotype [42]. Regardless whether ATM is able to phosphorylate these proteins and KU-55933 able to inhibit or not, ATM does not seem to function the same way in hESCs as in somatic cells. Furthermore, regardless whether classical NHEJ occurs in hESCs or not, our results suggest that repair is largely, if not completely, DNA-PKcs-independent. Recently, the role of DNA-PKcs in NHEJ in mouse and human ESCs was examined [8]. Whereas it was clearly demonstrated that in mouse ESCs DNA-PKcs is important for NHEJ it is unclear whether this is also the case in hESCs.

Since astrocytes seem to lack HRR, it should be possible to use these cells for examining casual relationships of proteins exclusively required for NHEJ. Indeed, we show that ATM kinase activity is strictly required for the formation and maintenance of repair foci in these cells. If KU-55933 was added prior to irradiation, a decrease of almost 90% in the number of γ-H2AX foci was seen, whereas when KU-55933 was added to cells after the formation of foci, they disassembled rapidly. There is a remote possibility that this represents faster than normal repair, however, this is highly unlikely since IRIF are not seen if the drug is added before irradiation. Furthermore, many, smaller IRIF remained in the KU-55933-treated cells which were only noticeable at high-power magnification. These foci were not resolved even at later times and eventually outnumbered the foci seen in the untreated control. Our results suggest that ATM plays a critical role in the formation and maintenance of repair foci in astrocytes. Furthermore, treatment of astrocytes with KU-57788 in part inhibited the rate of IRIF resolution. This finding indicates that while DNA-PKcs is involved to some extent in the repair of DSBs, ATM is vital for the initial signaling, formation and maintenance of repair complexes necessary for efficient DSB repair.

In summary, we have demonstrated that hESCs depend on HRR to a large extent whereas astrocytes do not and instead appear to exclusively utilize repair other than HRR. Importantly, hESCs requires ATR for HRR and not ATM, whereas astrocytes critically require ATM to keep repair factors assembled at DSBs and facilitate DSB repair. If astrocytes use classical NHEJ it is largely DNA-PKcs dependent. The contribution of NHEJ to DSB repair in hESCs is currently being investigated.

## Materials and Methods

### Antibodies and reagents

Antibodies used were anti-Oct3/4, -β-actin (Santa Cruz Biotechnology), -Nestin, -γ-H2AX (clone JBW301), -GFAP, -Sox2, -Musashi-1 (Millipore), -RAD51 (EMD Biosciences), -pT68-CHK2 (Cell Signaling), and -ATR (2B5), and -BrdU (Abcam). KU-55933 and KU-57788 (kindly provided by KuDOS Pharmaceuticals, Inc.), were dissolved in DMSO. Caffeine (Sigma-Aldrich) was dissolved in water.

### Cell culture and treatments

The human ESCs BG01V (ATCC, Rockville, MD) and H9 were cultured and differentiated on a feeder free system. BGO1V cells are embryonic stem cells that are easier to culture and more stable than BGO1 cells without inadvertently causing cell differentiation. They are a derivative of BG01 cells with karyotypic abnormalities (49, +12, +17 and XXY) which retain embryonic stem cell markers and characteristics, and the ability to differentiate down a neural lineage [43]. The H9 cells have a normal karyotype. Differentiation was performed to obtain NPs and astrocytes according to published protocols to obtain populations of NPs and astrocytes (Figure S1) [18], [44]. Briefly, hESCs cells were cultured in ES medium as described in [18], [45] consisting of Dulbecco's modified Eagle's medium (DMEM)/F12 medium (GIBCO, Grand Island, NY) supplemented with 20% knockout serum replacement (KSR) (GIBCO), 2 mM L-glutamine, 0.1 mM non-essential amino acids, 50 U/ml penicillin, 50 µg/ml streptomycin, 0.1 mM β-mercapto-ethanol, and 5 ng/ml basic fibroblast growth factor (b-FGF) (R & D Systems, Minneapolis, MN). Prior to experiments, cells were expanded on dishes of lysed MEF feeders. Derivation of NPs was performed as described [45]. Briefly, cells were grown on laminin coated dishes in DMEM/F12 medium containing 15% FBS and 5% KSR followed by an additional 7 days in media containing (DMEM/F12, N2 supplement (GIBCO), penicillin/streptomycin, L-glutamine, 5 ng/mL of b-FGF, and 10 ng/mL leukemia inhibitory factor (LIF). The cells were cultured in DN2 media for an additional 7 days to obtain the NP population. NPs were propagated on poly-ornithine- and laminin-coated plates in Neurobasal A Medium (GIBCO) supplemented with B-27 (GIBCO), L-glutamine, and penicillin/streptomycin, 20 ng/mL b-FGF, and 10 ng/mL of LIF. Astrocytes were obtained by exposure of NPs to DMEM and FBS for 25 days on laminin-coated plates as described [18], [44].

### IRIF assay

The IRIF assay has been described previously [21]. Cells were grown on Lab-Tek (Naperville, IL) glass slides. After treatment, cells were fixed with 3% paraformaldehyde, permeabilized with 0.5% Triton-X-100 in phosphate-buffered saline (PBS) and blocked with 10% non-fat dry milk/0.5% goat serum/PBS prior to exposure to primary antibodies followed by secondary antibodies Alexa 488 goat anti-rabbit or goat anti-mouse 546 Fab fragment (Invitrogen) at 1∶500 dilution, and nuclei counter-stained with DAPI (1 µg/ml). Cells were imaged and analyzed using a Zeiss LSM 510 Meta imaging system in the Massey Cancer Center Flow Cytometry and Imaging Facility. Foci were quantified by manual counting and the Cell Profiler Software (www.cellprofiler.org).

### Western blotting

Western blotting was performed as described [21], [24]. Proteins were separated by SDS-PAGE and transferred to PVDF membranes which were exposed to antibodies at 1∶200–2000 dilutions. Protein bands were detected and quantified using infrared emitting-conjugated secondary antibodies; anti-rabbit IRDYE 800 (Rockland Immunochemicals, Gilbertsville PA) or anti-mouse Alexa 680 (Invitrogen) using the Odyssey infrared imaging system from Li-Cor Biosciences (Lincoln, NE). Densitometry of immunoreactive band was performed using the Odyssey Application Software version 1.2 from Li-Cor Biosciences (Lincoln, NE).

### Down-regulation of ATR

ATR expression was down-regulated using Smart Pools L003202-00-0005 and M-003202-05 and GFP (control) 5′-GAACGGCAUCAAGGUGAACdTdT-3′ siRNA (Dharmacon, Inc.). siRNAs were transfected 24 hr after seeding of hESCs with 80 or 200 nM of siRNA using Dharmafect 1 or Amaxa nucleofection (Lonza AG) according to the manufacturer's instructions.

### Real-time qPCR

Genomic DNA was extracted using the High-Pure PCR Template Preparation Kit (Roche). Amplification of genomic DNA was performed on an ABI 7900HT Real-time qPCR instrument using Taqman Gene Expression Master Mix (Applied Biosystems). Relative ATR levels were determined after normalizing to GAPDH. The PCR primers used for ATR were HS00169878_m1 and for GAPDH were HS99999905_m1 (Applied Biosystems).

### Statistics

Unpaired two tailed t-tests were performed on triplicate or more data sets using GraphPad Prism 3.0 (GraphPad Software, Inc). P-values are indicated as *<0.05, **<0.01, ***<0.001, ns = non significant.

## Supporting Information

Figure S1 BG01V cells differentiate down a neural lineage to form neural progenitors and terminally differentiated astrocytes. (A) hESCs, (B) NPs and (C) astrocytes were stained with antibodies against the indicated differentiation markers. Top row in each set shows staining without DAPI and the bottom row shows the same fields with nuclear staining by DAPI. BG01V cells were routinely propagated with >95% of the cells expressing Oct3/4, Nestin, and Sox2 nuclear staining, suggesting that they maintained their embryonic stem cell character. hESCs were treated to obtain NPs characterized by >95% of cells expressing Nestin and Musashi-1, and >90% of them expressing Sox2. Astrocytes were isolated and characterized by ∼95% of cells expressing GFAP and also being negative for Oct3/4, Musashi-1, Sox 2, and Nestin. Less than 5% of the cells stained positive for the neuronal marker βIII-tubulin (picture is not representative of % positive cells). No cells showed positive staining for the marker O1 (oligodendrocyte specific). Less than 4% of the cells proliferated (BrdU+), suggesting that >96% of this population was terminally differentiated astrocytes (data not shown). These cell populations were used in the subsequent DSB repair studies.
(6.22 MB TIF)

Figure S2 (1.20 MB TIF)
