# Supplementary figures and images for: Dynamic Dependence on ATR and ATM for Double-Strand Break Repair in Human Embryonic Stem Cells and Neural Descendants
Source: PLoS One. 2010 Apr 2;5(4):e10001. doi: 10.1371/journal.pone.0010001 (PMC2848855; doi:10.1371/journal.pone.0010001)

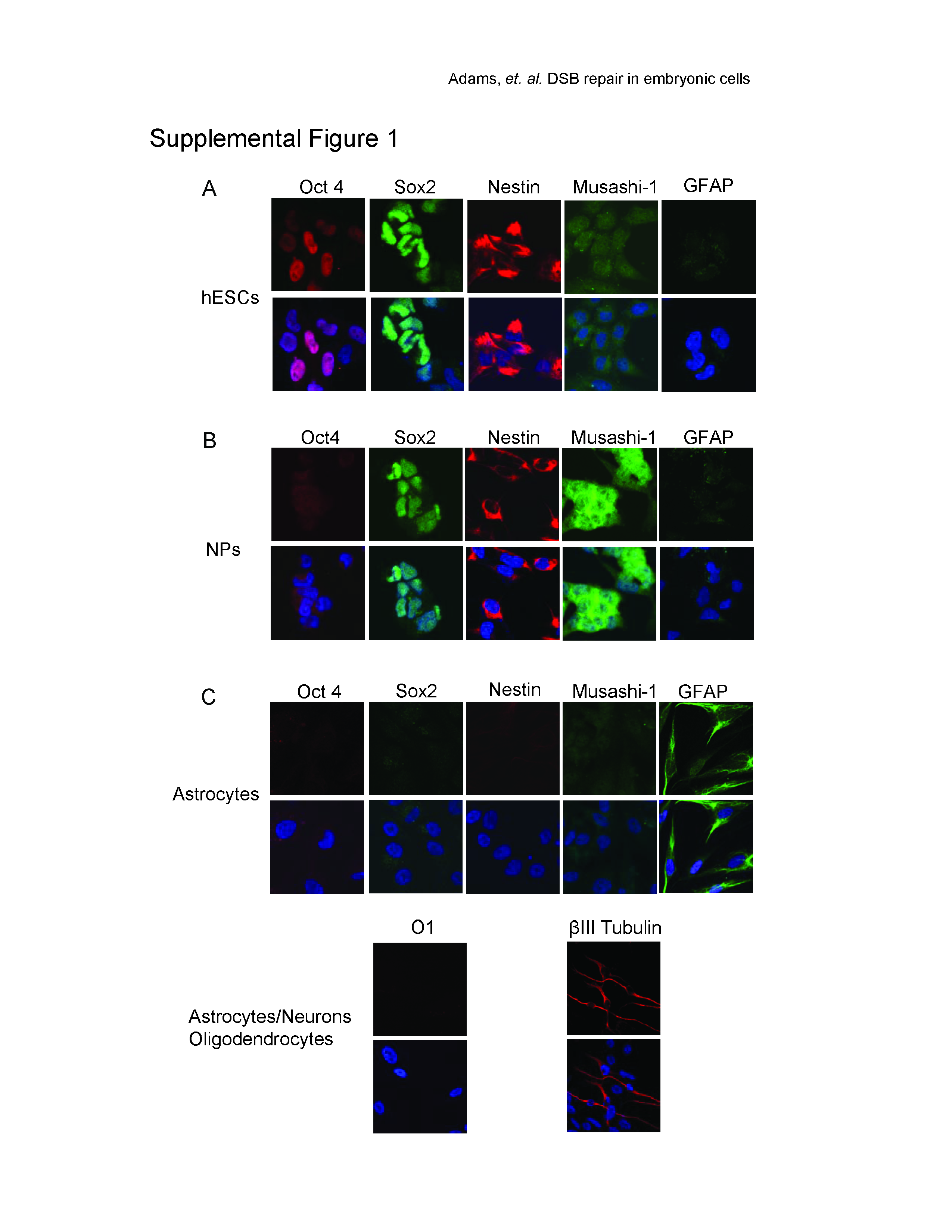

Supplement: Figure S1 — BG01V cells differentiate down a neural lineage to form neural progenitors and terminally differentiated astrocytes. (A) hESCs, (B) NPs and (C) astrocytes were stained with antibodies against the indicated differentiation markers. Top row in each set shows staining without DAPI and the bottom row shows the same fields with nuclear staining by DAPI. BG01V cells were routinely propagated with >95% of the cells expressing Oct3/4, Nestin, and Sox2 nuclear staining, suggesting that they maintained their embryonic stem cell character. hESCs were treated to obtain NPs characterized by >95% of cells expressing Nestin and Musashi-1, and >90% of them expressing Sox2. Astrocytes were isolated and characterized by ∼95% of cells expressing GFAP and also being negative for Oct3/4, Musashi-1, Sox 2, and Nestin. Less than 5% of the cells stained positive for the neuronal marker βIII-tubulin (picture is not representative of % positive cells). No cells showed positive staining for the marker O1 (oligodendrocyte specific). Less than 4% of the cells proliferated (BrdU+), suggesting that >96% of this population was terminally differentiated astrocytes (data not shown). These cell populations were used in the subsequent DSB repair studies. (6.22 MB TIF) [file pone.0010001.s001.tif]

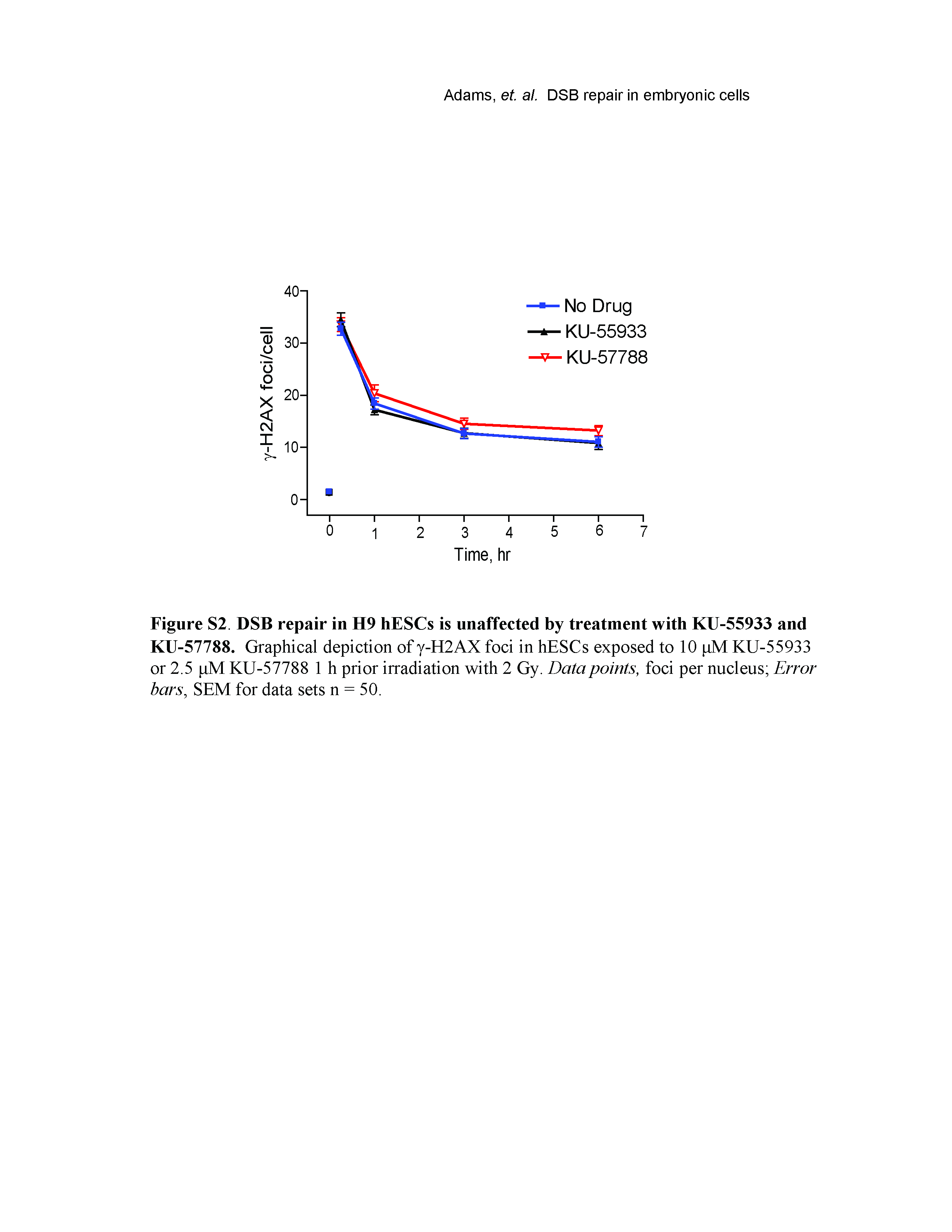

Supplement: Figure S2 — (1.20 MB TIF) [file pone.0010001.s002.tif]
